# Improving the *N*-glycosylation occupancy of plant-produced IgG1 by engineering the amino acid environment at Asn297

**DOI:** 10.3389/fpls.2024.1531710

**Published:** 2025-01-22

**Authors:** Kathrin Göritzer, Valentina Ruocco, Ulrike Vavra, Shiva Izadi, Omayra C. Bolaños-Martínez, Thareeya Phetphoung, Nuttapat Pisuttinusart, Waranyoo Phoolcharoen, Richard Strasser

**Affiliations:** ^1^ Department of Applied Genetics and Cell Biology, BOKU University, Vienna, Austria; ^2^ Department of Pharmacognosy and Pharmaceutical Botany, Faculty of Pharmaceutical Sciences, Chulalongkorn University, Bangkok, Thailand

**Keywords:** monoclonal antibodies, *Nicotiana benthamiana*, IgG1, *N*-glycosylation, glycosylation efficiency

## Abstract

Monoclonal antibodies are crucial recombinant biopharmaceuticals, with *N*-glycosylation at Asn297 essential for their functionality. Plants are increasingly used for antibody production, achieving high expression levels and enabling glycoengineering to produce homogenous human-like *N*-glycan structures. However, plant-produced human IgG1 often shows significant underglycosylation with potential adverse effects for immune functions and stability. This study addressed this limitation of the widely used plant-based expression platform *Nicotiana benthamiana* by employing protein engineering to enhance *N*-glycosylation occupancy in plant-produced IgG1. This was achieved through an amino acid mutation near the conserved glycosylation site in the CH2 domain of the heavy chain. The transient expression of trastuzumab and SARS-CoV-2 neutralizing IgG1 antibody COVA2-15 in *N. benthamiana*, with mutations such as Y300L, resulted in a notable improvement in glycosylation occupancy. While the structural integrity and monodispersity of the IgG1 variant remained unaltered, an improvement in thermal stability was observed. Furthermore, functional assays showed that antigen binding and human hFcRn interaction were unaffected, while FcγRIIIa binding affinity increased. These findings demonstrate the potential of protein-engineering to enhance the quality and functionality of plant-produced IgG1 antibodies, making them comparable to mammalian-produced counterparts.

## 1 Introduction

Monoclonal antibodies represent the most important and fastest-growing class of recombinant biopharmaceuticals, utilized in various therapeutic settings (Crescioli et al., 2024). The conserved *N*-glycosylation at Asn297 of human IgG1 is crucial for its proper folding and functionality, particularly in terms of receptor interaction. Consequently, glycosylation is considered a critical quality attribute of recombinant antibodies, necessitating tight control to ensure effective antibody functions, avoid unwanted side effects, and facilitate the development of biosimilars (Reusch and Tejada, 2015).

Plants are increasingly being used for the production of recombinant biopharmaceuticals and have successfully expressed various classes of highly effective recombinant antibodies against viruses and human antigens (Ruocco and Strasser, 2022; Shanmugaraj et al., 2022; Eidenberger et al., 2023; Göritzer et al., 2024a). Expression levels of more than 1 g/kg for IgG antibodies are frequently achieved by transient expression in *Nicotiana benthamiana* plants, making this system economically attractive (Bendandi et al., 2010; Ridgley et al., 2023). Host glycoengineering enables the production of functional IgG antibodies with human-like complex *N*-glycan structures that lack core fucose residues, resulting in increased functional activities (Strasser et al., 2008; Forthal et al., 2010; Zeitlin et al., 2011; Stelter et al., 2020).

However, it has been demonstrated that the transient expression of recombinant human IgG1 antibodies in *Nicotiana benthamiana* leaves results in significant underglycosylation at Asn297 in the Fc domain, which is likely influenced by the local amino acid environment adjacent to the *N*-glycosylation site (Murray et al., 2015; Castilho et al., 2018). In contrast, recombinant IgG1 antibodies produced in mammalian cells are typically more than 99% glycosylated at Asn297 (Stadlmann et al., 2008). The observed underglycosylation at the conserved *N*-glycosylation site increases the heterogeneity of recombinant IgG1 antibodies, potentially leading to adverse effects on immune effector functions. Non-glycosylated (i.e., both heavy chains in the assembled antibody lack *N*-glycans) or hemi-glycosylated (i.e., one heavy chain is non-glycosylated while the other is glycosylated) IgG1 antibodies typically display reduced effector functions (Ha et al., 2011; Ju and Jung, 2014).

To address this issue, several strategies have been employed to increase *N*-glycosylation occupancy in plant-produced proteins. One approach involves the co-expression of oligosaccharyltransferase (OST) subunits from *Leishmania* such as LmSTT3D or LdOST to compensate limitations of the plant endogenous OST complex (Castilho et al., 2018; Beihammer et al., 2023). Here, we aimed to address the issue of underglycosylation in plant-produced IgG1 by exploring an alternative strategy to enhance *N*-glycosylation occupancy. We focused on mutating amino acids adjacent to the *N*-glycosylation site to improve glycosylation efficiency. We generated various variants of the monoclonal IgG1 antibody trastuzumab, which targets the breast cancer antigen HER2, as well as the SARS-CoV-2 neutralizing monoclonal IgG1 antibody COVA2-15 (Claret and Vu, 2012; Brouwer et al., 2020; Göritzer et al., 2024a). This was achieved by mutating single amino acids near the *N*-glycosylation sequon to resemble sequences found in other IgG isotypes, such as human IgG2, IgG3, IgG4, and mouse IgG2, which have been previously approved for human therapy. To verify the broader applicability, we analyzed the glycosylation status, structural integrity, thermal stability, and receptor binding properties of the mutated IgG1 glycosylation variants.

## 2 Material and methods

### 2.1 IgG1 expression vectors

The codon-optimized genes of the heavy chain (OP892522.1) and light chain (OP892523.1) required for expression of trastuzumab IgG1 in *N. benthamiana* were synthesized by GeneArt (Thermo Fisher Scientific, USA). Sequences for expression in *N. benthamiana* were flanked with the signal peptide from barley alpha-amylase (AAA98615) and the restriction sites XhoI and AgeI. The synthesized DNA was then amplified by PCR with the primers “Strings_7F (CTTCCGGCTCGTTTGACCGGTATG)/Strings_8R (AAAAACCCTGGCGCTCGAG)”, and the constructs were separately cloned into the AgeI/XhoI sites of the binary vector pEAQ-HT (Sainsbury et al., 2009).

Construction of expression vectors for pEAQ-COVA2-15 heavy chain and light chain have been described previously (Göritzer et al., 2024a).

To generate IgG1 variants, point mutations were introduced using QuikChange site-directed mutagenesis kit (Agilent Technologies) according to manufacturer’s protocols using the primes described in **Supplementary Table S1**.

For expression in leaf epidermal cells of *N. benthamiana*, the plasmids were introduced into *Agrobacterium tumefaciens* strain UIA143 (Strasser et al., 2008).

Mammalian cell produced trastuzumab IgG1 was kindly provided by Alois Jungbauer (da Silva et al., 2019).

### 2.2 Plant material and agroinfiltration

*N. benthamiana* plants were grown at 23°C under long-day conditions (i.e., 16 h light/8 h dark). Infiltration into leaves of 5-week-old *N. benthamiana* was done as previously described (Göritzer et al., 2017). Briefly, the respective *Agrobacteria* were grown in LB-medium overnight at 29°C. Bacteria were centrifuged, resuspended in infiltration buffer (10 mM MgSO_4_, 10 mM MES and 0.1 mM acetosyringone) and the suspension was used for infiltration. Agrobacteria suspensions of heavy-chain plasmids were infiltrated with a OD_600_ of 0.15, while Agrobacteria suspension with light-chain plasmids were infiltrated with an OD_600_ of 0.1. Recombinant proteins were expressed *in N. benthamiana* glycosylation mutant plants (ΔXT/FT) (Strasser et al., 2008). Infiltrated leaves were harvested 5 days post infiltration (dpi) and used for protein extraction as previously described (Göritzer et al., 2024a).

### 2.3 Antibody purification

Clarified leaf extracts were passed through columns packed with Pierce Protein A resin (Thermo Fisher, USA). Proteins were eluted with 0.1 M glycine pH 3.5, followed by the immediate addition of 10% (v/v) 1 M Tris-HCl pH 9.0 to neutralize the pH. Fractions containing the protein of interest were pooled and dialyzed against 1xPBS pH 7.4 at 4°C overnight using a dialyzing cassette with 10-kDa molecular weight cutoff (MWCO; Slide-A-Lyzer, Thermo Scientific, USA). Pooled and dialyzed protein fractions were concentrated using Amicon centrifugal filters with an MWCO of 30 kDa (Merck Millipore) and subjected to SEC on a HiLoad 16/600 Superdex 200 pg column (GE Healthcare, USA) equilibrated with 1xPBS pH 7.4 connected to an ÄKTA pure (GE Healthcare, USA) fast protein LC system.

### 2.4 Mass spectrometric analysis

For mass spectrometric analysis of IgG1 glycopeptides, 20 µg of purified protein was S-alkylated with iodoacetamide and digested in solution with trypsin (Promega, Austria). The digested samples were loaded on a nanoEase C18 column (nanoEase M/Z HSS T3 Column, 100Å, 1.8 µm, 300 μm × 150 mm, Waters), detected with an Orbitrap MS (Exploris 480, Thermo Fisher Scientific, Austria) and the obtained data analyzed using Skyline Version 22.2 software.

For intact protein analysis, around 2 µg of the protein solution was directly injected to a LC-ESI-MS system (LC: Agilent 1290 Infinity II UPLC). A gradient from 15 to 80% acetonitrile in 0.1% formic acid (using a Waters BioResolve column (2.1 x 5 mm) at a flow rate of 400 μL/min was applied (9 minutes gradient time). Detection was performed with a Q-TOF instrument (Agilent Technologies 6230B LC- TOFMS) equipped with the Jetstream ESI source in positive ion, MS mode (range: 100-3200 Da). Instrument calibration was performed using ESI calibration mixture (Agilent). Data was processed using MassHunter BioConfirm B.08.00 (Agilent) and the spectrum was deconvoluted by MaxEnt. Two blank runs (injection of 5 μL MS-grade water) were performed prior to the injection of the samples and a blank run was performed in between each sample in order to reduce carry-over from previous measurements/samples.

### 2.5 SDS-PAGE

For reducing or nonreducing SDS-PAGE a total of 5 μg of purified protein was loaded on a 10% gel and visualized with Coomassie Brilliant Blue staining.

### 2.6 Differential scanning fluorimetry

Differential scanning fluorimetry (DSF) was conducted using a CFX real-time PCR instrument (Bio-Rad Laboratories, Hercules, CA, USA) in 1×PBS buffer at pH 7.4 as previously described (Göritzer et al., 2024b). In short, monoclonal antibodies were diluted to a concentration of 1 mg/mL in the formulation buffer. SYPRO Orange Fluorescent Dye (Thermo Fisher Scientific, Waltham, MA, USA) was diluted 1000-fold from a 5000× concentrated stock to prepare the working dye solution in the formulation buffer before addition to the antibody samples. Thermal denaturation was initiated by gradually increasing the temperature from 25 to 95°C at a rate of 0.05°C/s. Fluorescence intensity measurements were recorded using the FRET channel. Automated data processing of thermal denaturation curves involved truncating the dataset to eliminate post-peak quenching effects. The first derivative approach to calculate *T*m was used. In this method, *T*m is the temperature corresponding to the maximum value of the first derivative of the DSF melting curve.

### 2.7 Dynamic light scattering

DLS measurements were performed as described previously with protein concentrations of 500 µg/mL in 1×PBS pH 7.4 supplemented with 0.05% Tween on a Malvern Zetasizer nano-ZS (Malvern Panalytical, Malvern, UK) in a 12 mL quartz cuvette (Göritzer et al., 2024b). Samples were measured at 25.0°C, and the LS was detected at 173° and collected in automatic mode. The mean values and SDs of the number weighted diameter were calculated from three measurements for each sample, and each reported value is an average.

### 2.8 OMNISEC

SEC-LS was used to characterize the recombinant expressed proteins in solutions relating to their purity, native oligomers or aggregates, and molecular weights as previously described (Göritzer et al., 2024a). Analyses were performed on an OMNISEC multidetector gel permeation chromatography (GPC)/SEC system equipped with a refractive index detector, a right-angle LS detector, a low-angle LS detector and a UV/visible light photodiode array detector (Malvern Panalytical, Malvern, UK). A Superdex 200 Increase 10/300 GL column (Cytiva, Marlborough, MA, USA) was used and equilibrated with Dulbecco’s PBS without Ca and Mg, P04-361000 (PAN-Biotech, Germany), as running buffer. Experiments were performed at a flow rate of 0.5 mL min−1 at 25°C and analyzed using OMNISEC software version 11.40 (Malvern Panalytical, Malvern, UK). Proper performance of the instrument was ensured by calibration and verification using the 200 mg Pierce BSA standard (Thermo Fisher Scientific). Before analysis, samples were centrifuged (16,000× g, 10 min) and filtered through 0.2 mm Durapore PVDF centrifugal filter(s) (MilliporeSigma, Burlington, MA, USA). A 100 µL volume of each sample was injected at a concentration of 1 mg/mL.

### 2.9 Antigen-binding ELISA

To determine the binding of the purified recombinant IgG1 to the antigens HER2 and SARS-CoV-2 RBD, ELISA plates coated with 100 ng/well purified RBD-His or 250 ng/well purified HER2 (kindly provided by Elisabeth Laurent, BOKU University, Vienna) as previously described (Göritzer et al., 2017; Göritzer et al., 2024a). For detection HRP-labeled anti-human IgG antibody was used (W4031, Promega, Austria). The EC_50_ was calculated in GraphPad Prism 9.0.

### 2.10 Receptor binding by surface plasmon resonance spectroscopy

Binding of IgG glycosylation variants to human FcRn was determined by surface plasmon resonance (SPR) in three replicates, using the Biacore T200 system (Cytiva) at 25°C. A Biacore CM5 Sensor Chip (Cytiva) was directly coated with 2.5 µg/mL of hFcRn (R&D Systems, 8639-FC-050, P55899) using an amine coupling kit (Cytiva, BR-1000-50) to approximately 80 response units (RU). PBS pH 6 supplemented with 0.05% Tween-20 was used as running buffer. Recombinant IgG1 were injected at 25-400 nM for 60 s and allowed to dissociate for 60 s. The chip was regenerated in PBS pH 7.4. The binding kinetics, *k*_on_ (1/Ms), *k*_off_ (1/s) and *K*_D_ (nM) were calculated from global fittings using a 1:1 binding model (BIAcore T2 Evaluation software).

For *in vitro* binding experiments to the extracellular domain (amino acids 17-208) of FcγRIIIa/CD16a was performed using a Biacore T200 (Cytiva), first the sensor chip surface was captured with anti-His antibody with the His Capture Kit (Cytiva) to a CM5 chip as described in the manufacturers’ protocol. The capturing of anti-His antibody reached 32000 RU. Secondly, immobilization of the His-tagged FcγRIIIa (V158 allotype, AcroBiosystems) on the chip surface was performed for 60s with a concentration of 1 μg/mL and a flow rate of 10 μL/min in HEPES-EP running buffer. 40 RU units were achieved. The immobilization step was previously optimized to avoid avidity effects using lower concentration of FcγRIIIa. Flow cell 2 remained unmodified and served as a reference cell for the subtraction of systematic instrument noise and drift. IgG binding curves were generated in multi-cycle kinetic experiments at five different concentrations in three independent runs ranging from 31.25 nM to 500 nM with 180 seconds association and 480 seconds dissociation time at a flow rate of 10 μL/min. After each run, surface regeneration was accomplished using 10 mM glycine, pH 1.7, for 120 seconds at a flow rate of 30 μL/min. The binding kinetics, *k*_on_ (1/Ms), *k*_off_ (1/s) and *K*_D_ (nM) were calculated from global fittings using a 1:1 binding model (BIAcore T2 Evaluation software).

## 3 Results

### 3.1 Mutating amino-acids adjacent to the *N*-glycosylation site improves site-occupancy in plant-produced human IgG1

**Figure 1:**
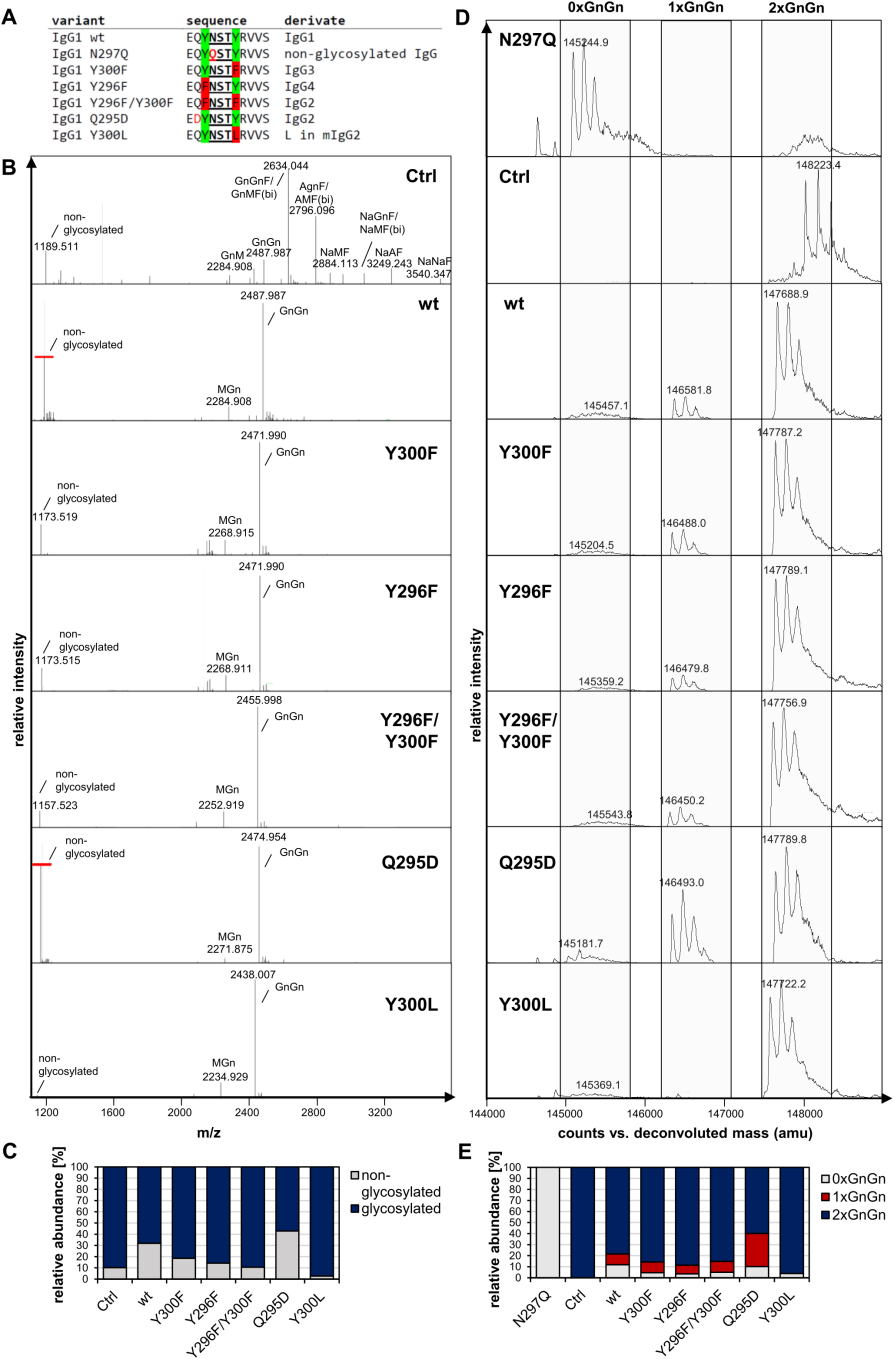
*N*-glycosylation analysis of trastuzumab (Tz) IgG1 variants produced in *N. benthamiana* ΔXT/FT. **(A)** Overview of the introduced amino acid sequence changes close to the conserved *N*-glycosylation site at position Asn297 of human IgG1. **(B)** LC-ESI-MS mass spectrometry spectra of tryptic peptide of Tz-IgG1 variants (wt:”EEQYNSTYR”, (M+2H)^2+^). Major glycoforms are labeled with abbreviations according to the ProGlycAn system (www.proglycan.com). **(C)** Quantification of the non-glycosylated glyco-peptide of Tz-IgG1 variants from LC-ESI-MS. **(D)** The *N*-glycan site occupancy of fully assembled intact Tz-IgG1 variants was determined using LC-ESI-MS. The peaks corresponding to non-glycosylated (0xGnGn), hemi-glycosylated (1xGnGn) and fully glycosylated (2xGnGn) IgG1 are highlighted. Multiple peaks represent different glycoforms and variations in the clipping of C-terminal lysine. **(E)** Quantification of the peaks from **D**.

We transiently expressed the monoclonal IgG1 antibody trastuzumab (“Tz”) in the glyco-engineered plant line *Nicotiana benthamiana* ΔXT/FT, which is almost completely devoid of β1,2-xylose- and α1,3-fucose-carrying *N*-glycans (Strasser et al., 2008). To investigate the role of the amino acid adjacent to the *N*-glycosylation sequon in glycosylation efficiency in plants, we generated various trastuzumab variants by mutating single amino acids near the *N*-glycosylation sequon of IgG1. These variants were designed to resemble sequences commonly found in other IgG isotypes, such as human IgG2, IgG3, IgG4, and mouse IgG2 (**Figure 1A**).

After transient expression of all IgG variants in *N. benthamiana* and small-scale purification from at least three pooled biological replicates, their glycosylation status was investigated. LC-ESI-MS analysis of the proteolytically digested heavy chain (HC) showed that, in contrast to commercially available trastuzumab produced in a mammalian cell line (“Tz-IgG_Ctrl”), considerable amounts of the non-glycosylated peptide are present in plant-produced wild-type IgG1 (~ 30%) and these amounts were further increased in the variant Q295D (~ 40%, **Figures 1B, C**). Other variants, including Y300F, Y296F and Y296F/Y300F, showed a notable reduction in the non-glycosylated peptide (~10-20%), with variant Y300L performing the best, which carries the mouse IgG2 sequence in close proximity to the sequon, exhibiting the most pronounced effect, with almost non-detectable amounts of non-glycosylated peptide. The reduction of underglycosylation was further demonstrated by immunoblotting of total soluble protein extracts under reducing conditions showing the presence of a double band representing the glycosylated (~ 53 kDa) and underglycosylated (~ 50 kDa) HC (**Supplementary Figure S1**), with a shift towards a single band for variant Y300L. This shift is caused by a higher degree of *N*-glycosylation occupancy as confirmed by Pngase F digestion followed by visualization by immunoblotting of Tz-IgG1 and variants Y300F and Y300L produced in *N. benthamiana* ΔXT/FT, which are not carrying α-1,3 linked core-fucose and can be cleaved by PngaseF in contrast to wild-type produced IgG1 (**Supplementary Figure S2**).

Besides the *N*-glycosylation efficiency of the different hosts, mammalian and plant-based systems also differ tremendously in terms of structural composition of attached *N*-glycans. The *N*-glycans found on plant-produced IgG1 variants showed a highly homogeneous profile, with biantennary complex-type GlcNAc2Man3GlcNAc2 (GnGn) as major glycoform. Mammalian cell-line produced trastuzumab glycan profiles are more complex and include high levels of galactosylation and sialylation, incomplete processing of branches and the possible modification with bisecting GlcNAc and core α1,6-fucosylation. Notably, the very homogenous glycosylation profile consisting of mostly GnGn type *N*-glycans of plant-produced IgG1 did not change by mutating amino-acids close to the *N*-glycosylation site demonstrating that the local amino acid environment does not affect complex *N*-glycan processing.

To further quantify and characterize the *N*-glycosylation occupancy of plant-produced IgG variants, we carried out an additional mass-spectrometry analysis by examining intact, fully assembled IgG (**Figures 1D, E**). While mammalian cell-line produced trastuzumab exhibits nearly complete *N*-glycosylation occupancy, intact MS measurements of the plant-produced wild-type IgG1 confirmed the presence of considerable amounts of non-glycosylated (0xGnGn) and the presence of hemi-glycosylated (1xGnGn) trastuzumab which is only glycosylated in one of the two HCs (together 24%). These values were significantly reduced in all variants, except for Q295D. Variant Y300L displayed the most favorable outcome, with up to 98% of the assembled antibodies being fully glycosylated (2xGnGn).

**Figure 2:**
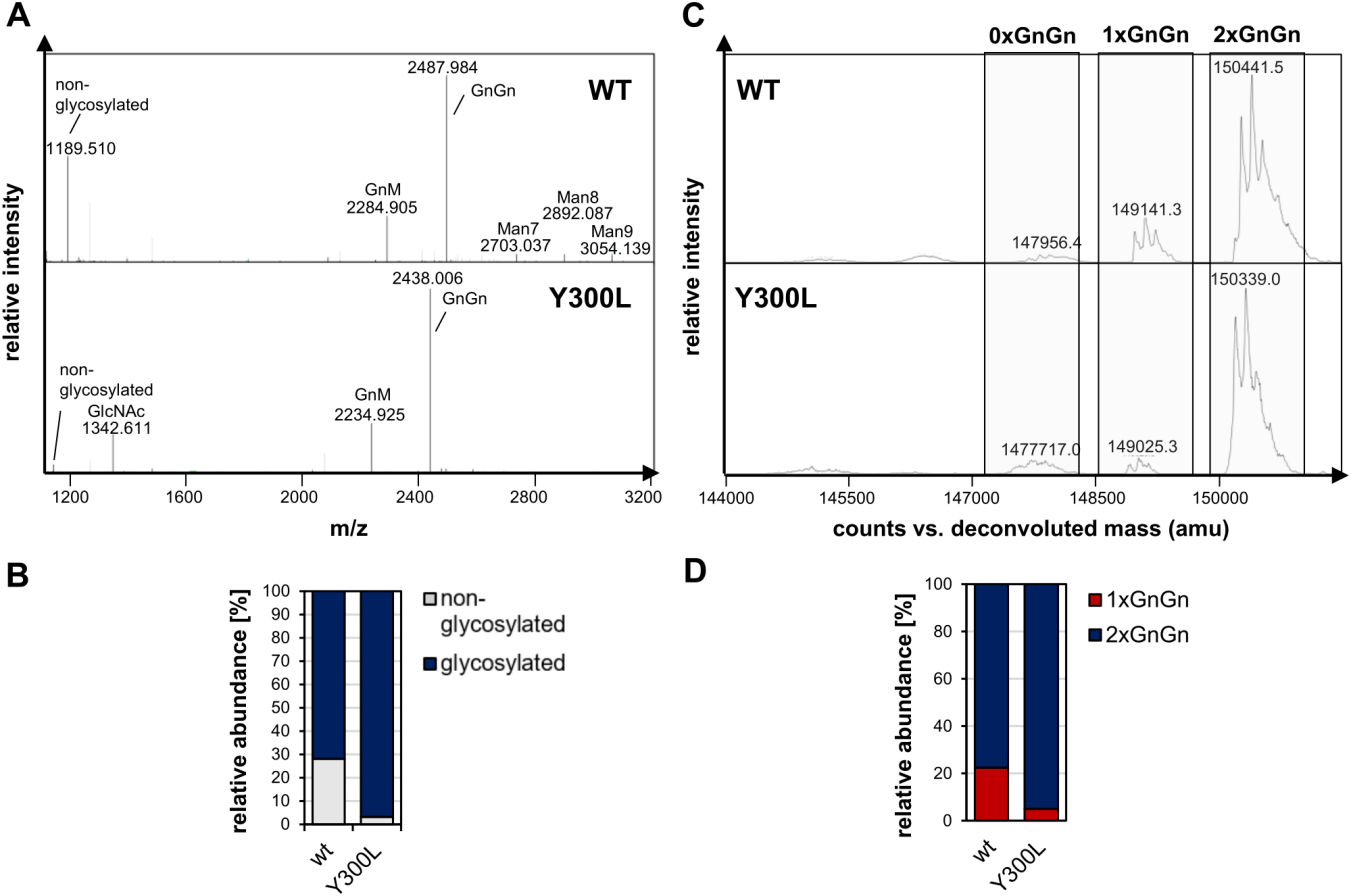
*N*-glycosylation analysis of COVA2-15 (CoV) IgG1 variants produced in *N. benthamiana* ΔXT/FT. **(A)** LC-ESI-MS mass spectrometry spectra of tryptic peptide of CoV-IgG variants (wt:”EEQYNSTYR”, (M+2H)^2+^). Major glycoforms are labeled with abbreviations according to the ProGlycAn system (www.proglycan.com). **(B)** Quantification of the non-glycosylated glyco-peptide of CoV-IgG variants from LC-ESI-MS from **A**. **(C)** The *N*-glycan site occupancy of fully assembled intact CoV-IgG variants was determined using LC-ESI-MS. The peaks corresponding to unglycosylated, hemi-glycosylated (1xGnGn) and fully glycosylated (2xGnGn) are highlighted. Multiple peaks represent different glycoforms and variations in the clipping of C-terminal lysine. **(D)** Quantification of the peaks from **C**.

To verify whether the Y300L mutation is also beneficial in terms of *N*-glycosylation occupancy in other monoclonal IgG1 antibodies, we generated a mutated variant of the SARS-CoV-2 neutralizing IgG1 antibody COVA2-15 (**Figure 2**). After transient expression in *N. benthamiana* and purification, wild-type and Y300L COVA2-15 IgG1 were analyzed via LC-ESI-MS of tryptic glycopeptides and subjected to intact mass spectrometry as described above. Glycopeptide analysis (**Figures 2A, B**) showed the presence of high amounts of non-glycosylated peptide in wild-type COVA2-15 IgG1. Intact MS measurements (**Figures 2C, D**) confirmed that underglycosylation is mostly the hemiglycosylated type (1xGnGn, ~22%). However, the Y300L mutation significantly improved *N*-glycosylation, reducing underglycosylation to approximately 5%.

### 3.2 IgG1 glycosylation variants exhibit similar overall structural integrity, but differ in thermal stability

**Figure 3:**
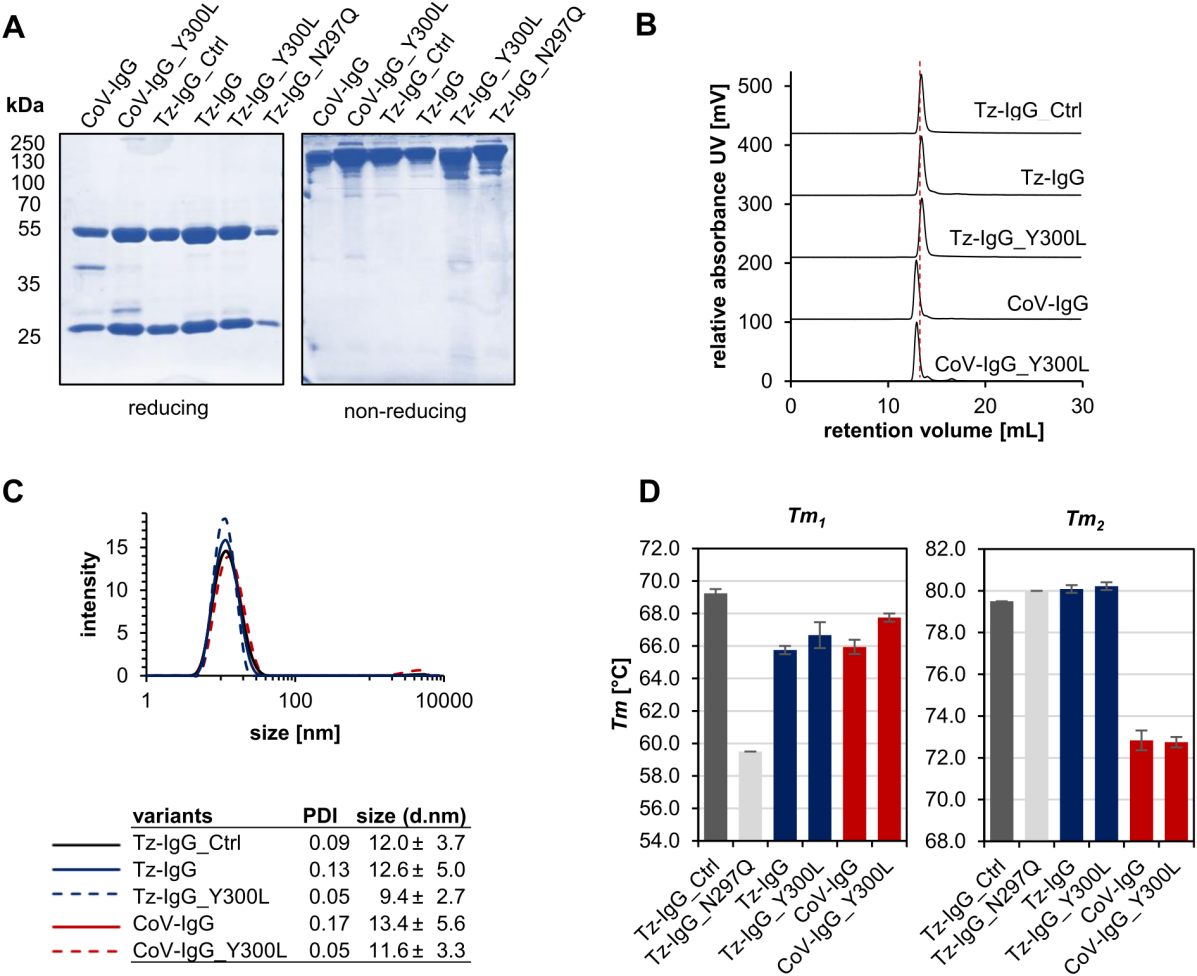
Characterization of purified recombinant IgG1 variants. **(A)** SDS-PAGE and Coomassie Brilliant Blue (CBB) staining of affinity and size-exclusion purified trastuzumab (Tz) and COVA2-15 (CoV) IgG1 variants from *N. benthamiana* ΔXT/FT. **(B)** Normalized size-exclusion chromatograms (A280) of affinity-purified and gel-filtration purified Tz- and CoV-IgG1 variants (A280 nm). **(C)** Dynamic light scattering (DLS) of Tz- and CoV-IgG1 variants. The mean diameter (size) and the homogeneity (PDI) were measured using a Malvern Zetasizer nano-ZS at 25°C. Each sample was measured in triplicates. **(D)** Thermal unfolding of Tz- and CoV-IgG variants was determined by differential scanning fluorimetry (DSF) in 1xPBS buffer pH 7.4. Experiments were performed at 1 mg/mL. Represented values are the mean ± SD of three technical repeats of three independent experiments.

In the next step, we scaled up the recombinant production of trastuzumab and COVA2-15 IgG1 variants in *N. benthamiana* to enable downstream analysis of the mutations’ effects and increased *N*-glycan occupancy on conformation and structural integrity. All variants were successfully expressed and purified, yielding amounts consistent with previously reported values (~100 mg/kg leaf fresh weight) (Kallolimath et al., 2020; Göritzer et al., 2024a). SDS-PAGE analysis of affinity- and SEC-purified IgG1 under reducing conditions showed predominant bands at the expected sizes of 53 kDa for the HC and 25 kDa for the light chain (LC) in all variants, except COVA2-15 IgG1. The latter displayed an additional minor band at around 40 kDa, corresponding to a commonly observed degradation product for this IgG1 variant in plants, likely due to proteolytic cleavage in the variable domain of the HC within the apoplast (manuscript under preparation; **Figure 3A**). Under non-reducing conditions all variants also showed characteristic bands at ~150 kDa corresponding to the fully assembled IgG1 antibody.

Size-exclusion chromatography coupled with light scattering revealed single, monodisperse peaks for all variants, with molecular sizes in the expected range of 149-151 kDa, confirming proper conformational integrity (**Figure 3B**; **Table 1**) and the absence of high molecular weight aggregates or additional degradation products. The high homogeneity of the variants was further supported by dynamic light scattering, which demonstrated a narrow particle size distribution. Notably, the Y300L mutants exhibited a significantly reduced polydispersity index (PDI < 0.05), indicating improved sample monodispersity compared to wild-type IgG1 produced *in planta* and in mammalian cell lines (**Figure 3C**). The non-glycosylated N297Q variant was not analyzed because it displayed a very high tendency to aggregate.

**Table 1:** Molecular weight (MW) and retention volume (RV) of recombinant IgG1 variants determined by SEC-LS.

	RV (mL)	MW (g/mol)
Tz-IgG_Ctrl	13.40	150539.5 ± 3889.4
Tz -IgG	13.41	148917.3 ± 4217.9
Tz -IgG_Y300L	13.47	149475.9 ± 3302.4
CoV -IgG	12.94	150851.8 ± 2384.0
CoV-IgG_Y300L	12.90	149559.1 ± 2099.3

Commercial trastuzumab (Tz-IgG_Ctrl) and *N. benthamiana* produced IgG1 variants were subjected to OMNISEC and molecular weight was determined with OMNISEC software v11.40 software.

We next performed differential scanning fluorimetry to assess the thermal stability of IgG1 variants. Thermal unfolding of all antibodies revealed two distinct transitions, corresponding to the unfolding of the Fc domain (Tm_1_) and the Fab domain (Tm_2_) (**Figure 3D**; **Supplementary Table S2**). While all variants showed similar *Tm*_2_ values to wild-type of around 80°C and 72°C for trastuzumab and COVA2-15, respectively, *Tm*_1_ varied significantly among the variants. Commercial trastuzumab exhibited the highest *Tm*_1_ (69.25°C), whereas the plant-produced non-glycosylated IgG1 (N297Q) displayed a 10°C reduction in thermal stability, consistent with previous reports highlighting the critical role of the Asn297 *N*-glycan in maintaining the IgG Fc domain’s conformation (Kiyoshi et al., 2017). Plant-produced wild-type trastuzumab IgG1 exhibited a *Tm*_1_ value 4°C lower than that of the commercial mammalian-produced version, emphasizing the role of not only the presence but also the composition of attached *N*-glycans for thermal stability of IgG1. Notably, the Y300L mutation improved the thermal stability of both trastuzumab and COVA2-15 IgG1, with an increase in *Tm*_1_ of up to 2°C compared to their wild-type counterparts further approaching values of mammalian produced IgG1.

### 3.3 Altered Fc glycosylation occupancy does not alter binding to antigens and has a minor impact on binding to the neonatal Fc receptor

**Figure 4:**
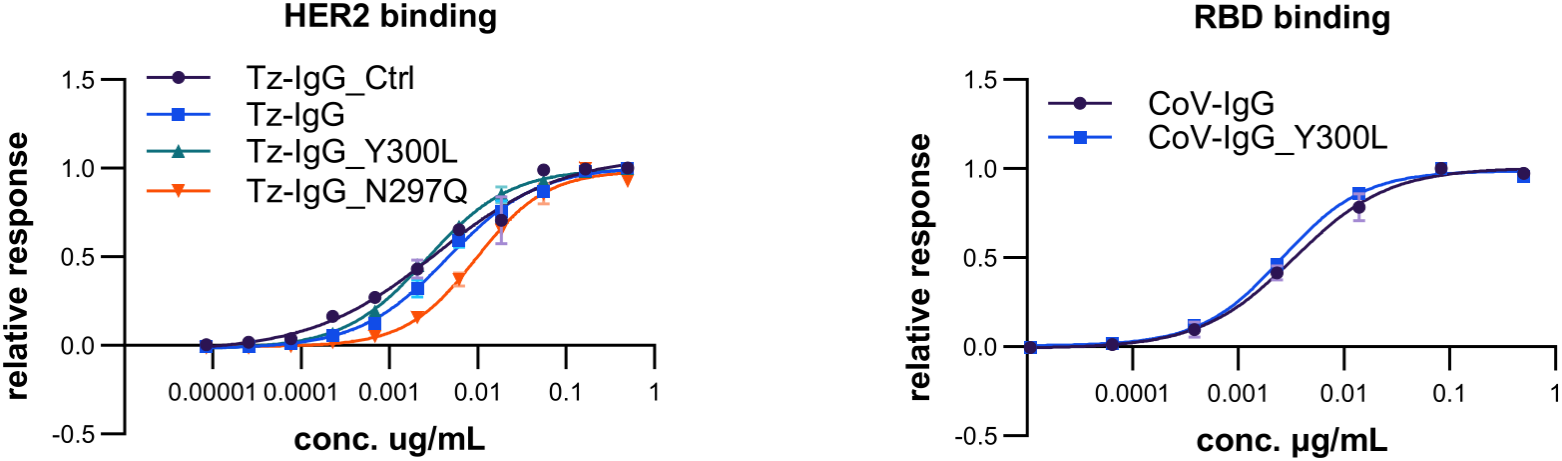
Antigen binding of different trastuzumab (Tz) and COVA2-15 (CoV) IgG1 variants. EC_50_ of binding of commercial trastuzumab (Tz-IgG_Ctrl) and recombinant Tz-IgG1 variants (Tz-IgG, Tz-IgG_N297Q, Tz-IgG_Y300L), as well as COVA2-15 IgG1 variants (CoV-IgG, CoV-IgG_Y300L) produced in *N. benthamiana* ΔXF plants to the respective antigens HER2 and SARS-COV-2 RBD was determined by ELISA. Each value is the mean ± SD from three independent measurements with two technical repeats and are depicted in detail in **Supplementary Table S3**.

We tested the functionality of the IgG1 glycosylation variants in terms of antigen-binding and receptor binding. Binding of trastuzumab and COVA2-15 IgG1 to the antigen HER2 and SARS-CoV-2 RBD, respectively, was unaffected by Fc-domain modifications (**Figure 4**; **Supplementary Table S3**). This is in accordance to previous reports that showed only minor cross-talk between the IgG1 Fc domain and antigen-binding capacities (Ha et al., 2011).

The human FcRn is a key determinant in extending the plasma half-life of antibodies by recycling them through transcytosis pathways (Pyzik et al., 2019). Binding of IgG glycosylation variants to FcRn was assessed using surface plasmon resonance (SPR). A Biacore CM5 sensor chip was coated with recombinant FcRn, and IgG variants were injected at concentrations ranging from 25 to 400 nM. Binding kinetics were analyzed using a 1:1 binding model to determine kinetic parameters, including the association rate constant (*k_on_*), dissociation rate constant (*k_off_*), and equilibrium dissociation constant (*K_D_*). Additionally, affinity was determined by steady-state kinetics (**Table 2**; **Supplementary Figure S3A**).

**Table 2:** Kinetic parameters of recombinant IgG1 binding to human FcRn determined by surface plasmon resonance (SPR) spectroscopy.

	*k_on_* (1/Ms)	*k_off_* (1/Ms)	*K_D_* (nM)	steady-state *K_D_* (nM)
Tz-IgG_Ctrl	1579899.1 ± 2.8x10^5^	0.084 ± 0.015	53.07 ± 0.77	195.31 ± 17.98
Tz-IgG_N297Q	124391.3 ± 2.9x10^4^	0.011 ± 0.001	93.47 ± 28.7	309.12 ± 33.87
Tz-IgG	701727.1 ± 8.9x10^3^	0.093 ± 0.042	94.00 ± 3.08	212.24 ± 1.49
Tz-Y300L	813348.6 ±1.5x10^4^	0.100 ± 0.051	84.21 ± 2.56	209.28 ± 4.62
CoV-IgG	389378.1 ± 9.6x10^3^	0.076 ± 0.001	195.57 ± 2.51	344.19 ± 28.14
COV-IgG_Y300L	170333.4 ± 2.4x10^4^	0.012 ± 0.001	74.36 ± 14.35	209.63 ± 9.82

Values for kinetic constants *k_on_*, *k_off_* and *K_D_*, as well as *K_D_* obtained by steady-state analysis represent means ± SD from three independent experiments at 5 different concentrations.

The interaction was characterized by fast association and very fast dissociation constants. Commercial trastuzumab (Tz-IgG_Ctrl) exhibited the highest *k_on_*, indicating rapid binding to FcRn, and the lowest *K_D_*, reflecting the highest affinity for FcRn. The association of plant-produced IgG variants was generally lower but improved with the introduction of the Y300L mutation. The dissociation constants were very fast for all variants and hence were challenging to fit in a 1:1 binding model. Therefore, affinity constants were also determined by steady-state. These *K_D_* values were generally higher than values determined by kinetic measurements and may be more reliable due to the rapid association and dissociation of the complex. Affinity determined by steady-state kinetics showed very similar FcRn binding of commercial and plant-produced trastuzumab, even when the Y300L mutation was introduced (**Table 2**; **Supplementary Table S5**).

The affinity of COVA2-15 IgG1 wild-type to FcRn was generally lower than the trastuzumab counterpart and was similar to the N297Q variant. Introduction of the Y300L mutation with increased *N*-glycosylation occupancy significantly improved FcRn binding, reaching the same affinity as commercial trastuzumab. The primary interaction between IgG1 and FcRn is mediated by the protein-protein interface involving the CH2 and CH3 domains of the Fc region, rather than directly by the *N*-glycans at Asn297. However, glycosylation at Asn297 contributes to the overall structural integrity and stability of the Fc region, which may indirectly influence binding. Indeed, the relatively low RU_max_ of the N297Q variant and the slightly reduced RU_max_ of COVA2-15 wild-type IgG1 compared to commercial trastuzumab (**Supplementary Table S4**) indicates the presence of aggregated or non-functional protein under the tested conditions, which is in accordance with the importance of the Asn297 *N*-glycan for the structural integrity of IgG1.

### 3.4 Improved *N*-glycan occupancy increases the binding to FcγRIIIa

It has been extensively shown that the *N*-glycan at position Asn297 significantly influences the binding affinity of IgG1 to Fcγ receptors (Reusch and Tejada, 2015; Golay et al., 2022). In this study, we investigated the binding of IgG1 variants to FcγRIIIa (CD16a, V158) using SPR (**Table 3**; **Supplementary Figure S3B**). An anti-His antibody was immobilized on a CM5 sensor chip, followed by the capture of FcγRIIIa and the injection of monoclonal antibodies at concentrations ranging from 31.25 to 500 nM. Binding kinetics were analyzed using a 1:1 binding model to determine kinetic parameters. For the interaction of IgG1 with FcγRIIIa, also a fast association and dissociation were characteristic, whereas better fits of the 1:1 model were achieved compared to binding interactions with FcRn as described above. Commercial trastuzumab exhibited the lowest affinity (*K_D_* =295.38 ± 4.31 nM), while plant-produced IgG1 variants showed a 3- to 4-fold increase in affinity to FcγRIIIa. This is in accordance with previous reports demonstrating the role of the α1,6-linked core fucose in the *N*-glycan at position Asn297 of mammalian-produced IgG1 in reducing the affinity to FcγRIIIa, which is significantly improved when this glycan moiety is removed, as in IgG1 produced in glyco-engineered *N. benthamiana* plants (Stelter et al., 2020). Improving the *N*-glycan occupancy through the Y300L mutation further increased binding affinity to FcγRIIIa for COVA2-15 IgG1 and to a lower extend for Trastuzumab IgG1 (**Supplementary Table S6**).

**Table 3:** Kinetic parameters of recombinant IgG1 binding to FcγRIIIa determined by SPR.

	*k_on_* (1/Ms)	*k_off_* (1/Ms)	*K_D_* (nM)	steady-state *K_D_* (nM)
Tz-IgG_Ctrl	131348.0 ± 462.7	0.0388 ± 0.0005	295.38 ± 4.31	501.92 ± 8.18
Tz-IgG	362784.9 ± 3811.0	0.0296 ± 0.0007	81.69 ± 2.21	166.80 ± 6.85
Tz-IgG_Y300L	425111.5 ± 1365.1	0.0325 ± 0.0002	76.47 ± 0.29	152.31 ± 6.02
CoV-IgG	306487.9 ± 2456.5	0.0388 ± 0.0007	126.74 ± 2.93	258.65 ± 17.57
CoV-IgG_Y300L	367319.4 ± 14973.9	0.0358 ± 0.0005	97.62 ± 5.49	194.99 ± 6.26

Values for kinetic constants *k_on_*, *k_off_* and *K_D_*, as well as *K_D_* obtained by steady-state analysis represent means ± SD from three independent experiments at 5 different concentrations.

## 4 Discussion

The production of recombinant IgG1 antibodies in plants, particularly in *Nicotiana benthamiana*, has been shown to result in significant underglycosylation at the conserved Asn297 site. This phenomenon has been consistently observed in both transiently produced IgG1 in *N. benthamiana* leaves and in stably expressed antibodies derived from various tissues and species (Rademacher et al., 2008; Vamvaka et al., 2016; Castilho et al., 2018; Jansing et al., 2019; Eidenberger et al., 2022). In contrast, mammalian cell-derived recombinant IgG1 is typically fully glycosylated at this site (Stadlmann et al., 2008).

The high expression levels achieved through viral vectors in plants can lead to an overload of the endogenous plant OST machinery, resulting in reduced *N*-glycosylation (Eidenberger et al., 2022). The limitation of the plant OST complex appears to be specific to distinct sites like Asn297 and is likely influenced by the local amino acid environment adjacent to the *N*-glycosylation site (Murray et al., 2015). While overexpression of the entire OST complex or individual subunits, such as LdOST or LmSTT3D, has been shown to increase *N*-glycan occupancy on recombinant glycoproteins expressed in plants, these approaches occasionally resulted in unwanted side effects (Castilho et al., 2018; Göritzer et al., 2020; Beihammer et al., 2023). For instance, the overexpression of LmSTT3D led to increased amounts of incompletely processed *N*-glycan structures, likely due to mislocalization to the Golgi apparatus and interference with other cellular processes (Castilho et al., 2018). LdOST seemed to be better suited and displayed only ER localization and did not lead to unwanted alterations of the overall *N*-glycan profile when co-expressed with recombinant antibodies (Beihammer et al., 2023).

To date, LdOST has only been employed for transient co-expression with plant-produced recombinant glycoproteins. For robust industrial-scale production of human IgG1 in *N. benthamiana*, the use of a stable engineered line expressing LdOST or, alternatively, the engineered Y300L IgG1 HC variants would be advantageous.

Our study aimed to address the issue of underglycosylation in plant-produced IgG1 by exploring strategies to enhance *N*-glycosylation occupancy through target protein engineering. We focused on mutating amino acids adjacent to the *N*-glycosylation site to investigate the role of the amino-acid sequence around the *N*-glycosylation sequon for glycosylation efficiency of the endogenous plant OST. We chose human sequences and sequences from murine immunoglobulins because human sequences are unlikely to cause adverse side effects, and several murine IgG1 and IgG2 antibodies, such as Ibritumomab tiuxetan, have been approved for human therapy (Grillo-López, 2002). The results demonstrated that mutating specific amino acids near the *N*-glycosylation sequon significantly improved glycosylation efficiency, while not changing the composition of attached glycans. Notably, the Y300L mutation resulted in almost non-detectable amounts of non-glycosylated peptide, indicating a substantial improvement in glycosylation occupancy.

Our findings suggest that protein engineering of the target protein, specifically through amino acid mutations adjacent to the *N*-glycosylation site, can lead to increased glycosylation efficiency. The Y300L mutation not only improved glycosylation occupancy, but also enhanced thermal stability of the IgG1 antibodies. This is consistent with previous studies that reported reduced thermal stability of non-glycosylated IgG1, primarily affecting the CH2 domain (Garber and Demarest, 2007).

While as expected antigen-binding was not affected, increased glycosylation of IgG1 also resulted in improved glycosylation-dependent activities, such as enhanced FcγRIIIa binding affinities. These results align with previous studies that reported decreased FcγRIIIa binding of hemi-glycosylated IgG1 (Ha et al., 2011). The recycling of human IgG1 via interaction with FcRn is a crucial factor in determining the serum half-life. It is established that FcRn interacts with specific amino acid residues located within the CH2 and CH3 domains, which are distant from the conserved *N*-glycosylation site (Ying et al., 2014). However, FcRn affinity chromatography showed differences between glycoengineered IgG1 variants with reduced binding of partially or fully deglycosylated variants (Cymer et al., 2017). The non-glycosylated trastuzumab variant displayed a reduced affinity for FcRn by SPR. Furthermore, a distinction in binding was identified between COVA2-15 IgG1 and the Y300L variant with increased *N*-glycosylation. Although the observed discrepancies in binding could be ascribed to variations in the aggregation propensity of the non-glycosylated IgG1 variants, it is conceivable that the glycans exert a more direct influence on FcRn binding.

Increasing the glycosylation occupancy in plant-produced IgG, combined with well-established glyco-engineering approaches in plants to produce monoclonal IgG antibodies with human-like *N*-glycosylation, can significantly enhance the performance of therapeutic plant-based IgG antibodies. These well-established approaches include producing *N*-glycans without core-fucose and with either terminal galactose or sialic acid, which are essential for enhancing the effector function and circulatory half-life of monoclonal antibodies (Strasser et al., 2009; Forthal et al., 2010; Castilho et al., 2015; Kogelmann et al., 2024).

In conclusion, our study demonstrates that targeted amino acid mutations near the *N*-glycosylation site can significantly enhance glycosylation efficiency in plant-produced IgG1 antibodies. This approach not only improves the structural integrity and thermal stability of the antibodies but also enhances their functional activities, such as receptor binding. These findings provide valuable insights into the optimization of plant-based expression systems for the production of high-quality recombinant antibodies.

## Data Availability

The original contributions presented in the study are included in the article/**Supplementary Material**. Further inquiries can be directed to the corresponding author.
