# Supplementary material for: Improving the N-glycosylation occupancy of plant-produced IgG1 by engineering the amino acid environment at Asn297
Source: Front Plant Sci. 2025 Jan 22;15:1531710. doi: 10.3389/fpls.2024.1531710 (PMC11794253; doi:10.3389/fpls.2024.1531710)
Supplement: Supplementary file 1 [file DataSheet1.docx]

**Supplementary Information**

**Improving the *N*-glycosylation occupancy of plant-produced IgG1 by engineering the amino acid environment at Asn297**

**Supplementary tables**

**Table S1: Forward (F) and reverse (R) primers used for side-directed mutagenesis of Tz- and CoV-IgG1 mutants.**

| **variant** | **primers** |
| --- | --- |
| Q295D | F CCTGTAGGTGCTGTTGTAATCTTCCTCTCTAGGCTTAGTC  R GACTAAGCCTAGAGAGGAAGATTACAACAGCACCTACAGG |
| Y296F | F CCTAGAGAGGAACAGTTCAACAGCACCTACAGG  R CCTGTAGGTGCTGTTGAACTGTTCCTCTCTAGG |
| N297Q | F CACAACCCTGTAGGTGCTCTGGTACTGTTCCTCTCTAGG  R CCTAGAGAGGAACAGTACCAGAGCACCTACAGGGTTGTG |
| Y300F | F CAGTACAACAGCACCTTCAGGGTTGTGTCTGTG  R CACAGACACAACCCTGAAGGTGCTGTTGTACTG |
| Y296F/Y300F | F CCTAGAGAGGAACAGTTCAACAGCACCTTCAGG  R CCTGAAGGTGCTGTTGAACTGTTCCTCTCTAGG |
|  |  |

**Table S2: Differential scanning fluorometry (DSF) of recombinant IgG1 variants**. Values represent means ± SD of at least three technical repeats of three independent experiments.

|  | ***T*_m1_ (°C)** | | | ***T*_m2_ (°C)** | | |
| --- | --- | --- | --- | --- | --- | --- |
| Tz -IgG_Ctrl | 69.25 | ± | 0.25 | 79.50 | ± | 0.00 |
| Tz -IgG_N297Q | 59.50 | ± | 0.00 | 80.00 | ± | 0.00 |
| Tz -IgG | 65.75 | ± | 0.25 | 80.08 | ± | 0.19 |
| Tz -IgG_Y300L | 66.67 | ± | 0.80 | 80.22 | ± | 0.19 |
| CoV-IgG | 65.94 | ± | 0.44 | 72.83 | ± | 0.47 |
| CoV-IgG_Y300L | 67.75 | ± | 0.25 | 72.75 | ± | 0.25 |

**Table S3: Binding of Tz- and CoV-IgG1 variants to HER2 and SARS-CoV-2 RBD, respectively.** EC_50_ of binding of commercial Trastuzumab (Tz-IgG_Ctrl) and Tz-IgG variants (Tz-IgG, Tz-IgG_N297Q, Tz-IgG_Y300L), as well as COVA2-15 IgG variants (CoV-IgG, CoV-IgG_Y300L) produced in *N. benthamiana* ΔXT/FT plants to the respective antigens HER2 and SARS-CoV-2 RBD was determined by ELISA. The mean ± SD of three separate experiments is shown.

|  | **EC_50_ (ng/mL) to HER2** | | |
| --- | --- | --- | --- |
| Tz-IgG_Ctrl | 3.396 | ± | 1.866 |
| Tz-IgG | 4.590 | ± | 0.887 |
| Tz-IgG_Y300L | 2.664 | ± | 0.28ß |
| Tz-IgG_N297Q | 9.564 | ± | 1.508 |
|  | **EC_50_ (ng/mL) to RBD** | | |
| CoV-IgG | 3.369 | ± | 0.943 |
| CoV-IgG_Y300L | 2.584 | ± | 0.398 |

**Table S4: RUmax of IgG1 variants binding to hFcRn using SPR.** The maximum binding response (RU_max_) is shown as the mean ± SD of three separate experiments.

|  | **RU_max_** | | |
| --- | --- | --- | --- |
| Tz-IgG_N297Q | 1.89 | ± | 0.23 |
| Tz-IgG_Ctrl | 36.22 | ± | 0.81 |
| Tz-IgG | 29.53 | ± | 0.46 |
| Tz-IgG_Y300L | 29.03 | ± | 0.74 |
| CoV-IgG | 17.49 | ± | 0.23 |
| CoV-IgG_Y300L | 15.52 | ± | 0.25 |

**Table S5: Statistical evaluation of IgG1 variants binding (steady-state *K*_D_) to hFcRn using in SPR**. Statistical analysis was performed using one-way ANOVA followed by Tukey’s multiple comparison test.

|  | ***K*_D_ (steady-state)** | |
| --- | --- | --- |
|  | **summary** | **p-value** |
| Tz-IgG_Ctrl vs. Tz -IgG_N297Q | *** | 0,0002 |
| Tz-IgG_Ctrl vs. Tz-IgG | ns | 0,8951 |
| Tz-IgG_Ctrl vs. Tz-Y300L | ns | 0,9496 |
| Tz-IgG_Ctrl vs. CoV -IgG | **** | <0,0001 |
| Tz-IgG_Ctrl vs. CoV-IgG_Y300L | ns | 0,9442 |
| Tz -IgG_N297Q vs. Tz-IgG | *** | 0,0007 |
| Tz -IgG_N297Q vs. Tz-Y300L | *** | 0,0006 |
| Tz -IgG_N297Q vs. CoV -IgG | ns | 0,3234 |
| Tz -IgG_N297Q vs. CoV-IgG_Y300L | *** | 0,0006 |
| Tz-IgG vs. Tz-Y300L | ns | >0,9999 |
| Tz-IgG vs. CoV -IgG | **** | <0,0001 |
| Tz-IgG vs. CoV-IgG_Y300L | ns | >0,9999 |
| Tz-Y300L vs. CoV -IgG | **** | <0,0001 |
| Tz-Y300L vs. CoV-IgG_Y300L | ns | >0,9999 |
| CoV -IgG vs. CoV-IgG_Y300L | **** | <0,0001 |

**Table S6: Statistical evaluation of IgG1 variants binding (*K*_D_) to FcγRIIIa using SPR**. Statistical analysis was performed using one-way ANOVA followed by Tukey’s multiple comparison test.

|  | ***K*_D_ (steady-state)** | | ***K_D_*** | |
| --- | --- | --- | --- | --- |
|  | **summary** | **p-value** | **summary** | **p-value** |
| Tz-IgG_Ctrl vs. Tz-IgG | **** | <0,0001 | **** | <0,0001 |
| Tz-IgG_Ctrl vs. Tz-Y300L | **** | <0,0001 | **** | <0,0001 |
| Tz-IgG_Ctrl vs. CoV -IgG | **** | <0,0001 | **** | <0,0001 |
| Tz-IgG_Ctrl vs. CoV-IgG_Y300L | **** | <0,0001 | **** | <0,0001 |
| Tz-IgG vs. Tz-Y300L | ns | 0,6120 | * | 0,0297 |
| Tz-IgG vs. CoV -IgG | **** | <0,0001 | **** | <0,0001 |
| Tz-IgG vs. CoV-IgG_Y300L | ns | 0,1026 | ** | 0,0078 |
| Tz-Y300L vs. CoV -IgG | **** | <0,0001 | **** | <0,0001 |
| Tz-Y300L vs. CoV-IgG_Y300L | * | 0,0110 | *** | 0,0010 |
| CoV -IgG vs. CoV-IgG_Y300L | *** | 0,0006 | **** | <0,0001 |

**Supplemental Figures**

**
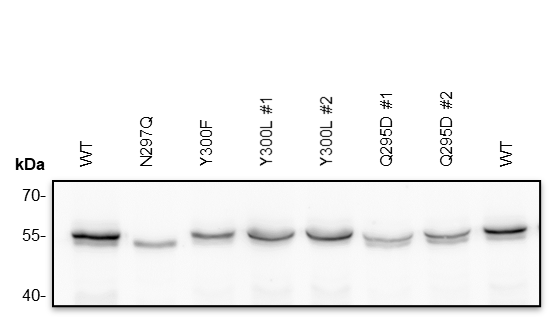
**

**Figure S1: Immunoblotting of Tz-IgG1 variants in crude protein extracts of *N. benthamiana* ΔXT/FT plants.** For visualization anti-IgG (anti-HC)-HRP antibody was used.


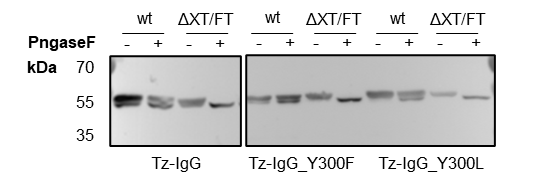


**Figure S2: Immunoblotting of Tz-IgG1 variants either produced *in N. benthamiana* wild-type (wt) or ΔXT/FT digested with PngaseF (+).** For visualization anti-IgG (anti-HC)-HRP antibody was used.


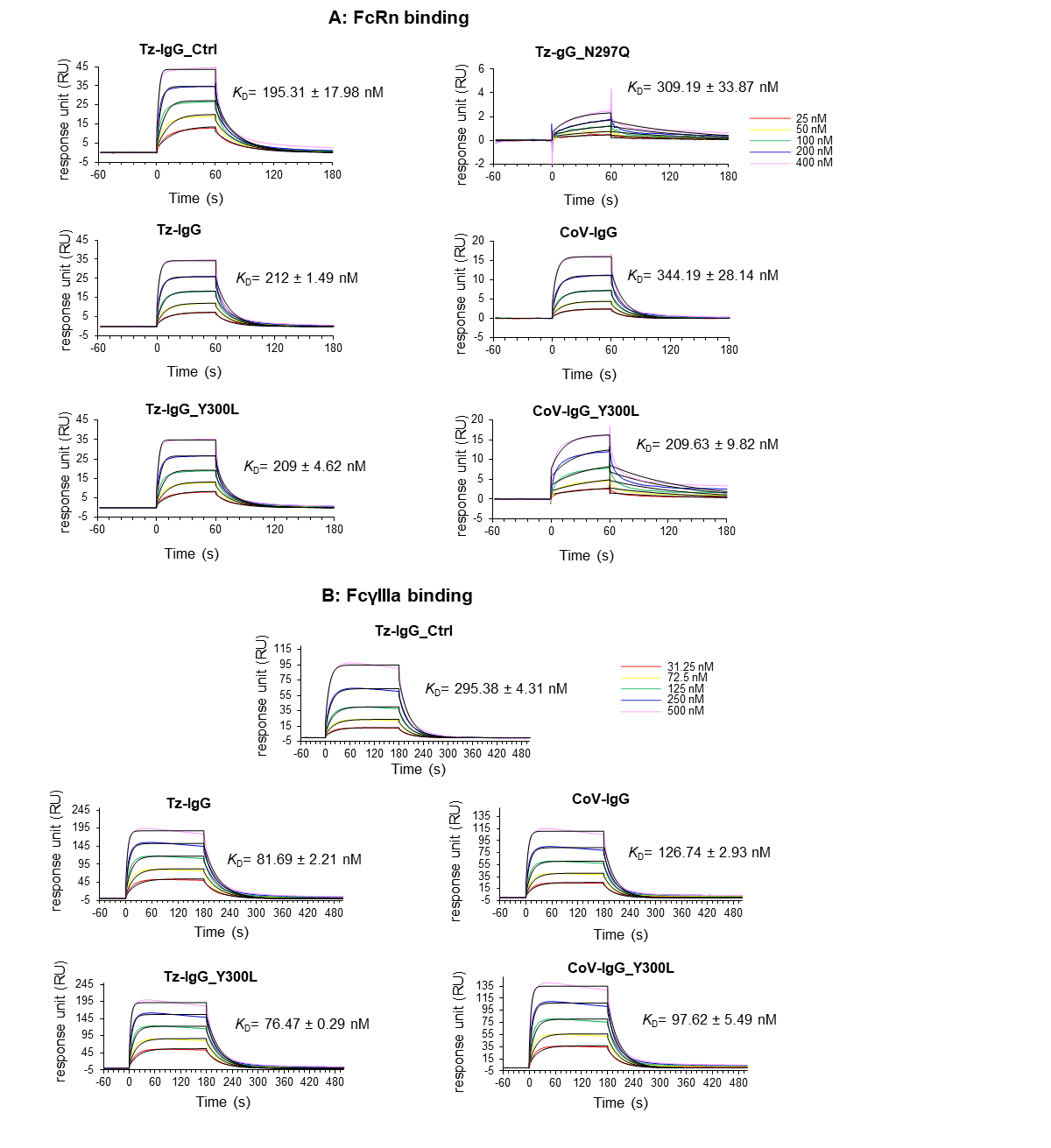


**Figure S3: Receptor binding of different trastuzumab (Tz) and COVA2-15 (CoV) IgG1 variants.** (**A**) SPR with multi-cycle kinetic experiments was used to compare the binding of FcRn to Tz- and CoV-IgG1 variants. The kinetic constants kon, koff and the equilibrium dissociation constants (KD), as well as the binding response (RU) are shown as the mean ± SD of three separate experiments. For more information also see Table 2 and S4. Sensorgrams exhibit five different concentrations ranging from 25 to 400 nM. Shown KD value was determined in steady state. (**B**) FcγRIIIa binding characteristics of Tz- and CoV-IgG1 variants was also determined by SPR spectroscopy in multi-cycle kinetic experiments from three independent measurements. Sensorgrams exhibit five different concentrations ranging from 31.25 to 500 nM. KD values depicted in the graph were determined from kinetic measurements as the mean ± SD.
